# Effect of *Bitis gabonica* and *Dendroaspis angusticeps* snake venoms on apoptosis-related genes in human thymic epithelial cells

**DOI:** 10.1590/1678-9199-JVATITD-2020-0057

**Published:** 2020-12-14

**Authors:** Francisc Boda, Krisztina Banfai, Kitti Garai, Bela Kovacs, Attila Almasi, Dalma Scheffer, Reka Lambertne Sinkler, Robert Csonka, Tamas Czompoly, Krisztian Kvell

**Affiliations:** 1 Department F1, Faculty of Pharmacy, George Emil Palade University of Medicine, Pharmacy, Science, and Technology of Targu Mures, Targu Mures, Romania.; 2 Department of Pharmaceutical Biotechnology, Faculty of Pharmacy, University of Pecs, Pecs, Hungary.; 3 Food Biotechnology Research Group, Szentagothai Research Center, University of Pecs, Pecs, Hungary.; 4 Department of Pharmaceutical Chemistry, Faculty of Pharmacy, University of Pecs, Pecs, Hungary.; 5 Soft Flow Ltd., Pecs, Hungary.

**Keywords:** Snake venoms, Bitis gabonica, Dendroaspis angusticeps, Apoptosis, Pyroptosis, Apoptosis mediators, RT-qPCR, Taqman array

## Abstract

**Background:**

Certain environmental toxins permanently damage the thymic epithelium, accelerate immune senescence and trigger secondary immune pathologies. However, the exact underlying cellular mechanisms and pathways of permanent immune intoxication remain unknown. The aim of the present study was to demonstrate gene expressional changes of apoptosis-related cellular pathways in human thymic epithelial cells following exposure to snake venom from *Bitis gabonica* and *Dendroaspis angusticeps.*

**Methods:**

Snake venoms were characterized by analytical methods including reversed phase high-performance liquid chromatography and sodium dodecyl sulphate-polyacrylamide gel electrophoresis, then applied on human thymic epithelial cells (1889c) for 24 h at 10 μg/mL (as used in previous TaqMan Array study). Gene expressional changes restricted to apoptosis were assayed by TaqMan Array (Human Apoptosis Plate).

**Results:**

The most prominent gene expressional changes were shown by *CASP5* (≈ 2.5 million-fold, confirmed by dedicated quantitative polymerase chain reaction) and *CARD9* (0.016-fold) for *B. gabonica,* and *BIRC7* (6.46-fold) and *CASP1* (0.30-fold) for *D. angusticeps.*

**Conclusion:**

The observed apoptotic environment suggests that pyroptosis may be the dominant pathway through which *B. gabonica* and *D. angusticeps* snake venoms trigger thymic epithelial apoptosis following envenomation.

## Background

Programmed cell death (PCD) represents an intracellular mechanism that leads to the death of cells through various pathways. PCD can be triggered by external factors, such as toxins, viruses or chemical agents, but also drives differentiation and developmental processes including regulation of cell number, deletion of unnecessary cell structures and elimination of dangerous cells. The three main forms of PCD are apoptosis, autophagy and programmed necrosis [1,2].

The cytotoxic and pro-apoptotic effect of snake venoms has been extensively studied due to the possible application of venom-derived components as anti-cancer agents [3-5]. The pro-apoptotic effect has been studied from venoms of numerous genera, such as *Bitis* [6], *Bothrops* [7-10], *Cerastes* [6,11], *Echis* [12], *Lachesis* [13], *Ophiophagus* [14], *Naja* [15,16] and *Walterinessia* [17]. Furthermore, the apoptosis inducing effect of individual components isolated from snake venoms has also been studied, demonstrating that L-amino acid oxidases (LAAO) [18-20], snake venom metalloproteinases (SVMP) [21,22], disintegrins [23,24] and phospholipases A_2_ (PLA_2_) [9,25] are capable of inducing apoptosis in various cell types. Although the apoptotic effect of snake venoms and their components are well characterized, there are still few reports on how these venoms affect other PCD mechanisms. Spermine, a polyamine isolated from the venom of *Eristicophis macmahonii* is capable of inducing autophagy in *Trypanosoma brucei*, demonstrated by the presence of autophagy vesicles and dilated mitochondria [26]. A recently published study reported that cytotoxin 1, a polypeptide isolated from *Naja atra* venom increases lysosome membrane permeability and the release of cathepsin B, meaning that this molecule likely induces necroptosis in leukemia cells [27]. Furthermore, the venom of the European honey bee (*Apis mellifera*) has been associated with increased caspase-1 and inflammasome activity [28], suggesting a possible induction of pyroptosis in treated cells. These observations suggest that animal venoms, including snake venoms are capable of inducing PCD through both apoptotic and non-apoptotic mechanisms.

Immune senescence, and within that thymic ageing develops in every person, although at individual rate. Thymic senescence is observed as adipose involution during which the thymus shrinks and functional epithelial network is replaced by adipose tissue [29]. Thymic ageing initiates during childhood to speed up during adolescence [30]. This process drastically impairs the production of naive T cells, cutting it by 90% by the age of 50 years in human, contributing to increased occurrence of infection, cancer and auto-immunity at senior ages [31-33]. Specific external factors may accelerate thymic senescence. Examples include chemicals (copper-chelators), hormones (androgens), infections (viruses, fungi, protozoa) and potentially biological toxins (e.g. snake venoms) [34-38]. Thymic senescence research will help to understand the underlying molecular- and cellular mechanisms and appoint new therapeutic targets for intervention strategies. Such interventions may slow down thymus senescence and enhance T cell production. This would in turn decrease the incidence of infection, cancer and auto-immunity that currently affects the senior population. In addition, gain in healthcare costs and quality of life share tremendous economic and social interest. 

Our study aimed to determine the effect of *Bitis gabonica* (*BG*) and *Dendroaspis angusticeps* (*DA*) venoms on a set of apoptosis-related genes using TaqMan Array technology in human thymic epithelial cells. Considering that these venoms originate from two different families of venomous snakes (Viperidae and Elapidae), a secondary objective was to evaluate similarities and differences concerning the effects of these venoms. Finally, based on our results, we proposed to highlight the possible long-term effect of envenomation at the cellular level with respect to thymic epithelial cells. Biological toxins (e.g. snake venoms) may trigger long-lasting alterations in the central immune system (thymus) through apoptotic pathways, thus accelerating immune senescence, with significant defects in health.

## Methods

### Snake venoms

Lyophilized *BG* and *DA* venoms were obtained from commercial sources (Latoxan S.A.S., Portes-les-Valence, France). Stock solutions were prepared by dissolving the lyophilized snake venoms in sterile phosphate buffered saline (PBS) (GE Healthcare Life Sciences, Chicago, IL, USA) at a final concentration of 10 mg/mL. Serial stock solutions were further diluted with sterile PBS to obtain the work solutions at concentrations of 30, 100, 300 and 1000 μg/mL.

### Characterization of snake venoms

Earlier methods for the purification, fractionation and characterization of the snake venom applied gel filtration and cation exchange without [39] or with high-performance liquid chromatography (HPLC) measurement [40]. The analysis itself was performed on reversed phase HPLC (RP-HPLC) chromatography on C-18 column, mainly with gradient (or partly isocratic) methods. A common feature of these analyses was that these venoms were initially dissolved and eluted in neutral 0.1 M ammonium-acetate buffer or 0.1 M NaCl, and the constant components of the acetonitrile and water with 0.1 % trifluoroacetic acid (TFA). The combination of the RP-HPLC and sodium dodecyl sulfate-polyacrylamide gel electrophoresis (SDS-PAGE) was already mentioned as a characterization method in the earlier publications [41], although improved RP-HPLC and SDS-PAGE profiling methods appeared later. Recently, the combination of RP-HPLC and SDS-PAGE represents a general strategy for snake venom analysis [42,43]. In order to characterize our *BG* and *DA* venoms, a Knauer HPLC equipped with C18 column (250 mm × 4.6 mm, 5 µm particle size, YMC) was used. Eluents were HPLC grade acetonitrile (ACN) and water with 0.1 % TFA. Gradient of 5% ACN for 5 min, 5-15% ACN over 20 min, 15-45% ACN over 120 min, and 45-70% ACN over 20 min were applied with 1 ml/min flow speed. Detector wavelength was set to 215 nm. 2 mg lyophilized venom was dissolved in 200 µl ACN-H_2_O (5%), 0.1% TFA and injected to a 20 µl loop. Separated elution peaks were lyophilized directly from the eluent. For SDS-PAGE analysis the fractions were reconstituted in water, mixed with 2x loading buffer (125 mM TrisHCl pH 6.8, 4% SDS, 20% glycerol, 100mM DTT), denatured for 5 min at 100˚C, and were separated on 15% SDS-polyacrylamide gel. Proteins were visualized by Coomassie-blue staining. For evaluation, we have applied a molecular weight marker/protein ladder of 10 to 180 kDa (Thermo Scientific, Pageruler Prestained Protein Ladder, Cat. No: 26616).

### Cell line

Human thymus-derived 1889c thymic cell line was cultured in RPMI 1640 medium (Lonza, Basel, Switzerland) supplemented with 10% fetal bovine serum (EuroClone, Pero, Italy), 2 mM L-Glutamine (Lonza, Basel, Switzerland) and penicillin (100 U/mL) - streptomycin (100 U/mL) mixture (Lonza, Basel, Switzerland). Cells were grown at 37 °C in a 5% CO_2_ atmosphere. Cell viability was assessed prior to treatment using an EVOS XL Core Cell Imaging System (Invitrogen, Carlsbad, CA, USA).

### **Incubation of cultured 1889c cells with *BG* and *DA* venoms**

The optimal incubation period of 1889c cell cultures with unfractionated *BG* and *DA* venoms has been established by carrying out a set of experiments, to evaluate both early and late changes in gene expression. In the first experiment, 1889c cell cultures were treated with 0.3, 1.0, 3.0 and 10 μg/mL *BG* or *DA* venom solutions, followed by incubation at 37 °C in a 5% CO_2_ atmosphere for 2 hours. In the second experiment, thymic epithelial cells were treated with 0.1, 1.0 and 10 μg/mL *BG* or *DA* venom solutions, followed by incubation in identical conditions, but for 24 hours. The selection of the concentration range was based on previous experiences involving snake venoms and TaqMan Array technology, an experiment during which different concentrations of snake venoms solutions have been used on cell cultures, resulting in the observation that relatively high venom concentrations are required to induce a measurable effect in gene expression of *ex vivo* cell cultures [44]. Cells incubated without treatment served as negative control in both experiments. All treatment conditions (venom type, venom concentration, incubation time) were plated in triplicates, and cell cultures with identical treatments were pooled following incubation. The effect of different venom concentrations and incubation duration was determined by microscope evaluation and RT-qPCR (real-time quantitative polymerase chain reaction) analysis. Cell confluence was assesses by microscope image analysis using ImageJ software 1.51j8 (Additional file 1).

### RNA isolation and cDNA synthesis

Total RNA was isolated from all cell cultures using a NucleoSpin RNA II kit (Macherey-Nagel, Düren, Germany) according to the manufacturer’s instructions. The concentration of the isolated total RNA was determined using a Nanodrop 2000 spectrophotometer (Thermo Fisher Scientific, Waltham, MA, USA). The isolated RNA was reversed transcribed to cDNA with an Applied Biosystems 2720 Thermal Cycler system (Applied Biosystems, Foster City, CA, USA) and the High-Capacity cDNA Reverse Transcription Kit (Applied Biosystems, Foster City, CA, USA) following on the manufacturer’s protocol.

### RT-qPCR analysis of selected apoptosis associated genes

The effect of *BG* and *DA* venoms on the expression of *BAD*, *BAX*, *CDKN1A* (encoding p21), *TP53* (encoding p53) and *BCL2* genes (IDT, Integrated DNA Technologies, Leuven, Belgium) (for primer list see Table 1) was determined using a Quantstudio 12K Flex Real-Time PCR System (Applied Biosystems, Foster City, CA, USA) with SensiFAST SYBR Hi-ROX Mix (Bioline, London, UK). Gene expression was normalized to *HPRT1* housekeeping gene and the results analyzed using Expression Suite Software (version 1.1., Thermo Fisher Scientific, Waltham, MA, USA).

**Table 1.** List of primer sequences used for RT-qPCR analysis.

Gene name	Primer sequence
BAD-for	GAGGTCCTGAGCCGACAG
BAD-rev	CTTCCTCTCCCACCGTAGC
BAX-for	AAGAAGCTGAGCGAGT
BAX-rev	GCCCATGATGGTTCTG
BCL2-for	CATCTCATGCCAAGGGGGAA
BCL2-rev	ATTCTTGGACGAGGGGGTGT
CDKN1A-for	CTGGGGATGTCCGTCAGAAC
CDKN1A-rev	CATTAGCGCATCACAGTCGC
HPRT1-for	CTGGCGTCGTGATTAGTGAT
HPRT1-rev	ACATCTCGAGCAAGACGTTC
TP53-for	CGCTTCGAGATGTTCCGAGA
TP53-rev	CTTCAGGTGGCTGGAGTGAG

### TaqMan Array of apoptosis associated genes

The expression of an extensive number of apoptosis associated genes was determined using a TaqMan Array Human Apoptosis, Fast 96-well Plate (Part No. 4418717, Applied Biosystems, Foster City, CA, USA). The TaqMan Array Plate contained 92 assays for apoptosis associated genes and 4 assays for endogenous control genes. The RT-qPCR amplification was performed on a Quantstudio 12K Real-Time PCR System (Applied Biosystems, Foster City, CA, USA) in a 10 μL final volume, containing TaqMan Fast Advanced Master Mix (2x) (Applied Biosystems, Foster City, CA, USA) and cDNA samples from cells treated with 10 μg/mL *BG* venom, 10 μg/mL *DA* venom or untreated cells (negative control). Gene expression was analyzed using Expression Suite Software (version 1.1., Thermo Fisher Scientific, Waltham, MA, USA).

## Results

### Snake venom analysis and selection

The composition and activity of snake venoms show significant variation among major and minor taxonomic groups along with intra-species differences due to age, diet or geographic location. Among these, the phylogenetic variations are the most relevant [45]. Elapid venoms are characterized by the presence of small molecular weight toxins, such as three-finger toxins and PLA_2_s, while viperid venoms predominantly contain high molecular weight enzymes including SVMPs, LAAOs, serine-proteases and PLA_2_s [45]. Consequently, we proposed to evaluate the composition and apoptosis-triggering activity of representatives of the family Elapidae (*Dendroaspis angusticeps)* and the family Viperidae (*Bitis gabonica)*. 

**Figure 1.**
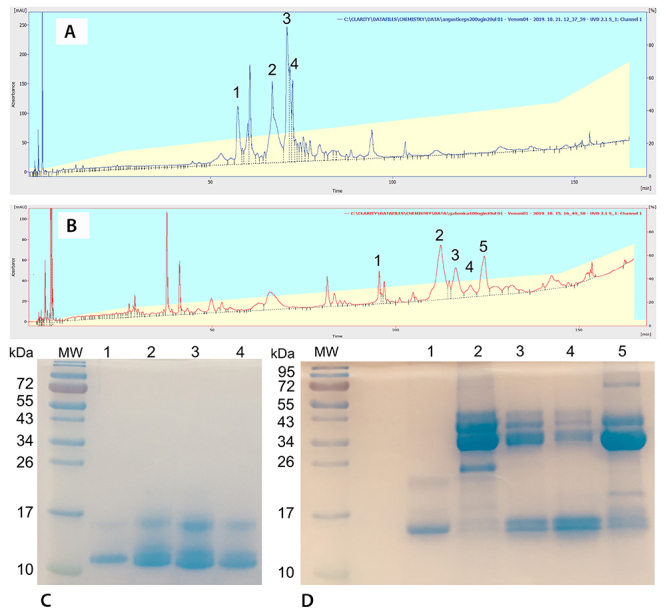
Chromatogram obtained following HPLC separation of **(A)** *Dendroaspis angusticeps* venom with four main fractions; **(B)** *Bitis gabonica* venom with five main fractions. All of these fractions have been isolated and further analysed by SDS-PAGE. Gel images show protein fractions separated based on molecular weight for **(C)** *D. angusticeps* and **(D)** *B. gabonica*. Lane numbers correspond to fraction labels isolated through HPLC separation, **(A, C)** 1-4 for *D. angusticeps* and **(B, D)** 1-5 for *B. gabonica*. The first lane on each gel contains Thermo Scientific Pageruler Prestained Protein Ladder 10 to 180 kDA (Cat. No.: 26616).

RP-HPLC analysis of the tested snake venoms showed characteristic and well-observable peaks. In case of *DA* several peaks were identified in the timespan of 50-100 minutes of the 180-minute analysis time (Figure 1A). In a prorated view the chromatograms presented by Petras et al. [46] show a similar pattern. Although using a different HPLC chromatographic system, major elution peaks were centered between approx. 30-60 minutes, having a total analysis time of 120 minutes. Further similarity is observed in the pattern of chromatographic records. In the middle region of peak elution four large peaks are distinguished in both analyses and several smaller ones can also be found at higher elution times. In our study we isolated and further analyzed the most prominent proteins by SDS-PAGE (Figure 1C). Our results show that low-molecular weight proteins (10-20 kDa) are characteristic of our venom fraction. These findings are in harmony with literature data of Petras et al. [46], identifying these proteins as short- and long acting neurotoxins and dendrotoxins. Results published by Conlon et al. [47] using similar protein characterization also found low-molecular weight F-VIII toxins in the venom of *Dendroaspis angusticeps*, with putative anti-tumor activity.

In contrast, HPLC run of *BG* venom showed a wider distribution of components throughout the 180-minute elution time (Figure 1B). Similarly, the SDS-PAGE analysis revealed the presence of low molecular weight proteins from 15 kD to the more abundant region of 30-50 kDa (Figure 1D). The analysis of *BG* venom showed several similarities with results published by Calvete et al. [48]. Along with a similar HPLC chromatographic pattern, SDS-PAGE analysis of the fractionated venom also revealed the presence of low-molecular weight proteins varying from 6.5 to 200 kDa. In a study conducted by Calvete et al. [49] the general characterization of *Bitis* species unveiled a heterogeneous composition comprising PLA_2_ molecules, C-type lectin-like proteins, LAAOs, serine proteases and different classes of SVMPs. In the 30-50 kDa region serine-proteases and SVMPs are typical representatives accounting for 50% of the total venom proteins, as published by Calvete et al. [49]. In addition, C-type lectins and PLA_2_ make up for 10-15% and may also be considered as major protein components of *BG* snake venom. The presence of these protein families predispose for PCD, and make *BG* venom a more destructive candidate for cell demise, in comparison with *DA* venom.

### Effect of incubation time and venom concentration

Prior to performing experiments, a systematic review of available methods has been performed, to assess the possible incubation time and venom concentration needed to induce apoptosis, without leading to untimely cell death. Based on this 2-hour (short) and 24-hour (long) incubation times have been selected, with venom concentrations ranging from 0.3 - 10 μg/mL to 0.1 - 10 μg/mL, respectively. Although the selected time points and concentrations might not overlap with lethal doses or therapeutic concentrations, these parameters were appointed for use in Petri-dish cultured cells in our experiments. The selected concentration also correspond to previously reported values [44,50,51].

As 1889c cell cultures were treated with *BG* and *DA* venoms for 2 hours and 24 hours, this allowed for the evaluation of both early and late changes in gene expression. During the 2-hour incubation 1889c cells have been treated with 0.3, 1.0, 3.0 and 10 μg/mL *BG* or *DA* venom solutions, while the 24-hour incubation has been carried out with 0.1, 1.0 and 10 μg/mL venom solutions. All treatment conditions were plated in triplicates, and cell cultures with identical treatments were pooled following incubation. The overall effect of snake venoms has been determined by microscope evaluation of the cell cultures. In comparison with untreated cells (negative control), all treatment conditions showed a decrease in the number of visible cells. Following the 2-hour incubation period, even at the highest concentration used, only a small number of apoptotic-resembling cells were visible (showing apoptotic blebs or vesicles). In contrast, the effect of snake venoms after 24-hour incubation was more pronounced, with a significant decrease of visible cells and the more frequent appearance of apoptotic-resembling cells, especially at the highest concentration of venoms used (10 μg/mL). Representative microscope images showing the observed effects are shown in Additional file 2.

Further evaluation of the effect of incubation time and venom concentration was performed using conventional low-throughput RT-qPCR analysis. The selected genes are closely related to apoptotic signaling pathways including *BAD, BAX, BCL2, CDKN1A* (encoding p21) and *TP53* (encoding p53). Gene expression was normalized to *HPRT1* housekeeping gene, while untreated 1889c cells served as reference. Table 2 shows mean fold change of genes, using relative quantity (RQ) values.

**Table 2.** Relative quantity (RQ) values of gene expression measured 2-hour and 24-hour after treatment of 1889c cells with *BG* or *DA* venom. Treatment conditions were plated in triplicates then pooled following incubation. Untreated cells served as reference (baseline).

Sample	RQ values of target genes					
Venom	Incubation time (h)	Conc. (μg/mL)	*BAD*	*BAX*	*CDKN1A*	*TP53*
*BG*	2	0.3	0.916	0.999	1.077	1.116
		1.0	0.872	0.989	1.109	1.076
		3.0	0.868	0.999	1.133	1.087
		10	0.937	1.093	1.414	1.166
	24	0.1	0.142	0.074	0.436	1.222
		1.0	0.515	0.664	1.043	0.946
		10	0.405	0.703	1.866	0.822
*DA*	2	0.3	0.867	0.850	1.193	0.817
		1.0	0.805	0.887	1.545	0.801
		3.0	0.767	0.837	1.823	0.760
		10	0.765	0.867	2.120	0.707
	24	0.1	0.806	0.941	1.108	0.893
		1.0	0.784	0.867	1.056	0.777
		10	1.402	1.019	1.677	0.767

The measured RQ values showed a positive correlation between incubation time and venom concentration for two genes encoding pro-apoptotic mediators (*BAD, CDKN1A*), while the expression of *BCL2* gene did not show a measurable change in any condition. Based on these results and the microscope images discussed previously, samples incubated for 24 hours with 10 μg/mL *BG* or *DA* venom have been selected for further high-throughput TaqMan Array analysis.

### Gene expression array in 1889c cells treated with snake venoms

Gene expression was assayed using the TaqMan Array Human Apoptosis Plate containing 92 apoptosis associated genes and four endogenous control genes allowing for normalization (*18S, GAPDH, GUSB* and *HPRT1*). Our data suggest a significant effect on gene expression, as almost all the studied genes showed alteration compared to baseline. The complete list of genes with the obtained RQ values is shown in Additional file 3.

For the purpose of this work, we have selected genes that presented changes over log2 RQ ≥ ±1.0 values (+10% tolerance limit giving >1.8 RQ or <0.55 RQ). Based on these criteria, 16 genes have been identified in the *BG* treatment group and 10 genes in the *DA* treatment group.

### **Gene expression in 1889c cells treated with *BG* venoms**

**Figure 2.**
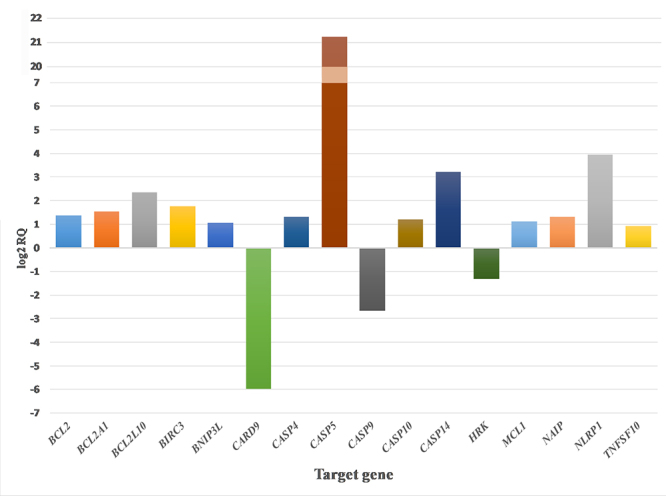
Up- and downregulated genes in 1889c cells treated with 10 μg/mL of *BG* venom. Values are shown using a log2 RQ-based scale. Untreated cells served as baseline (represented by value 0 of Y axis). The +1 and -1 values represent a two-fold increase or decrease threshold.

1889c cells treated with *BG* venom expressed a significant variation in the fold change of 16 genes associated with apoptosis (Figure 2). The most significant change (approx. 2.5 million-fold increase) was observed in the case of *CASP5*, the gene encoding inflammatory protease caspase-5. Since the fold change measured for this gene was unusually high, a dedicated conventional RT-qPCR measurement has also been performed for *CASP5* expression and confirmed this value. Other up-regulated genes of the caspase gene group included *CASP4* (2.47-fold increase), encoding inflammatory protease caspase-4, *CASP10* (2.30-fold increase), encoding apoptosis initiator caspase-10 and *CASP14* (9.40-fold increase), encoding caspase-14, also known to be involved in keratinocyte differentiation. On the other hand, the treatment resulted in the down-regulation of *CASP9*, the gene encoding caspase-9 (RQ = 0.15).

Members of NOD and leucine-rich repeat containing proteins gene group showed a significant increase in expression level. The *NLRP1* gene, encoding NLRP1, showed a 15.52-fold increase, while *NAIP*, the gene encoding NLR family apoptosis inhibitory protein (NAIP) presented a 2.49-fold increase.

A significant effect of *BG* venom was observed on genes of the apoptosis regulator Bcl2 family. The assessed up-regulated genes encoding anti-apoptotic Bcl2 family members included *BCL2* (2.56-fold increase), *BCL2A1* (2.88-fold increase), *BCL2L10* (5.08-fold increase), and *MCL1* (2.17-fold increase). Simultaneously, the gene *BNIP3L*, encoding the pro-apoptotic mediator Bcl2/adenovirus E1B 19 kDa protein-interacting protein 3-like (BNIP3L) displayed a 2.07-fold increase, while *HRK*, encoding the activator of apoptosis harakiri (HRK) protein presented a 0.4-fold decrease in gene expression. 

Other noteworthy findings include the up-regulation of *BIRC3* (3.40-fold increase), the gene encoding baculoviral IAP repeat containing protein 3 (BIRC3) and *TNFSF10* (1.90-fold increase), encoding TRAIL. Furthermore, *CARD9*, the gene encoding the CARD9 protein, displayed a 0.016-fold decrease in its expression.

### **Gene expression in 1889c cells treated with *DA* venom**

**Figure 3.**
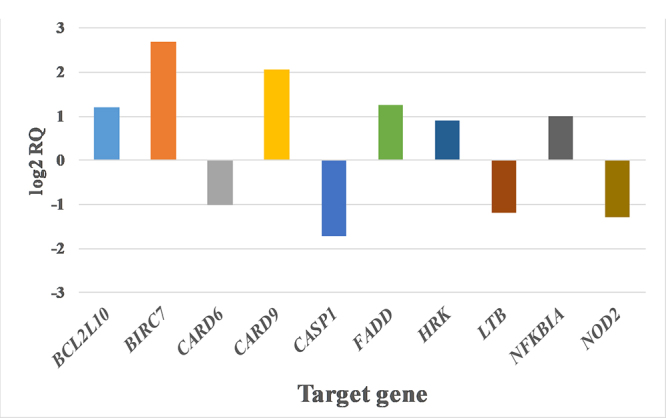
Up- and downregulated genes in 1889c cells treated with 10 μg/mL *DA* venom. Values are shown using a log2 RQ-based scale. Untreated cells served as baseline (represented by zero value of Y axis). The +1 and -1 values represent the two-fold increase or decrease threshold.

The treatment of 1889c cells with *DA* venom led to the significant variation in the expression of ten genes associated with apoptosis (Figure 3). Among these genes, the most relevant up-regulation has been observed in the case of *BIRC7* (6.46-fold increase), the gene encoding apoptotic mediator BIRC7. Two members of the Bcl2 family have been up-regulated, namely *BCL2L10* (2.3-fold increase) and *HRK* (1.86-fold increase). Further up-regulated genes included *CARD9* with a 4.13-fold increase, *FADD* with a 2.39-fold increase and *NFKBIA* with a 1.98-fold increase. The *FADD* gene encodes the pro-apoptotic mediator FADD, while *NFKBIA* encodes nuclear factor kappa-light-chain-enhancer of activated B cells (NF-κB) inhibitor alpha (IκBα).

Down-regulated genes included *CARD6* (0.49-fold decrease), *CASP1* (0.30-fold decrease), *LTB* (0.44-fold decrease) and *NOD2* (0.40-fold decrease). These genes encode CARD6, caspase-1, lymphotoxin beta and NOD2, respectively.

## Discussion

### Overview of programmed cell death mechanisms

Apoptosis, or type I PCD, plays an essential role in the selective removal of cells that are either damaged or no longer required during development or growth [52,53]. Apoptosis is characterized by distinct morphological changes, such as cell contraction, nuclear fragmentation, membrane bleb formation and loss of adherence [1]. The defective function of apoptotic mechanisms can lead to an accumulation of undesired cells in the tissues, resulting in cancer development [54], as well as autoimmune [55] or inflammatory diseases [56]. On the other hand, excessive apoptosis can cause tissue damage [53] or even severe neurodegenerative diseases [57].

There are two distinct pathways by which apoptosis can be triggered [53,58]. The intrinsic (mitochondrial) pathway involves mitochondrial outer membrane permeabilization (MOMP), regulated by pro- and anti-apoptotic Bcl-2 family members [2]. MOMP leads to the release of mitochondrial proteins, including cytochrome C. This binds to the apoptotic protease activating factor 1 (APAF1), leading to the formation of the apoptosome complex [59]. The apoptosome cleaves pro-caspase-9, generating active caspase-9, a major initiator of mitochondrial apoptosis, that in turn activates caspase-3 [1,60].

The extrinsic (death receptor) pathway of apoptosis is primarily triggered through membrane-bound death receptors (DR) that belong to the tumor necrosis factor (TNF) superfamily [53]. These include TNF receptor 1 (TNFR1), Fas receptor (FasR), death receptor 3 (DR3), TNF-related apoptosis-inducing ligand (TRAIL) receptor 1 (TRAILR1, DR4), TRAIL receptor 2 (TRAILR2, DR5) and death receptor 6 (DR6) [1,61]. The binding of mediators containing a death domain (DD) to these receptors initiates the signal for apoptosis. Examples include TNF-α and TNFR1, Fas ligand (FasL) and FasR, TNF-like protein 1 (TL1A) and DR3, and between TRAIL and TRAILR1/2 [1,^53^,^62^]. Ligand-receptor binding induces the recruitment of adaptor proteins with a death-effector domain (DED), such as Fas-associated death domain (FADD) or TNFR-associated death domain (TRADD) [53,63]. These in turn recruit pro-caspase-8 and pro-caspase-10, forming the death-inducing signaling complex (DISC) and activate the ligated caspases. Caspase-8 and caspase-10 function as initiator caspases and are responsible for the cleavage and activation of effector caspases, e.g. caspase-3, caspase-7 [62,64]. 

Autophagy, or type II PCD, is the process of self-degradation of cellular components associated with nutrient deprivation, pathogen infection and other intra- or extracellular stress sources [65]. Through autophagy, cellular components, protein aggregates, damaged organelles and invasive pathogens are degraded by the formation of autophagosomes. [2]. While the main function of autophagy is the promotion of cell survival under extreme conditions, an over-activation of autophagic mechanisms may result in cell death [66,67]. 

As the primary role of autophagy is the promotion of cell survival, it is capable of suppressing apoptosis. However, in some cases, autophagy leads to cell death, either in association with apoptosis, or as a secondary mechanism if the apoptotic pathways are inhibited. As such, there is a relevant and extensive crosstalk between pathways related to autophagy and apoptosis [2,67]. 

Regulated necrosis, or type III PCD, involves the swelling of organelles and cells, cell lysis and release of intracellular contents [1,68,69]. The most studied form of regulated necrosis is named necroptosis, which uses a unique signaling pathway, namely receptor-interacting protein kinase-1 and -3 (RIPK1, RIPK3), that may be specifically inhibited by necrostatins [68]. Known activators of necroptosis include TNF-α, FasL, TRAIL (initiators of the extrinsic apoptotic pathway), genotoxic stress, anticancer drugs, lipopolysaccharides, interferons, etc. [68,69].

Relatively well-known types of regulated necrosis include the mitochondrial permeability transition dependent regulated necrosis [69,70] and parthanatos, which relies on the activation of poly(ADP-ribose) polymerase 1 and the release of apoptosis-inducing factors [70-72]. Other forms of regulated necrosis include ferroptosis, an iron-dependent form of non-apoptotic cell death [73], pyroptosis, a caspase-1-dependent inflammatory cell death [74], and pyronecrosis, which requires cathepsin B and apoptosis-associated speck-like protein containing caspase recruitment domain [69,75].

Pyroptosis is a purposeful form of PCD, as regulated necrosis, in which inflammatory caspases, mainly caspase-1 (canonical pathway), -4, and -5 (non-canonical pathway) occupy a cardinal role, leading to the maturation of pro-inflammatory cytokines inside an inflammasome [76,77]. Initiator sensors like nucleotide-binding oligomerization domain (NOD), leucine rich repeat and pyrin domain containing-1 and -3 (NLRP1, NLRP3), and NLR (NOD-like receptor) family CARD (caspase activation and recruitment domain)-containing protein 4 (NLRC4) also play a role [78]. Novel insights concerning the underlying mechanisms of pyroptosis reveal an important role of gasdermin D, the primary effector molecule in pyroptosis [79,80]. To date, pyroptosis, as an important regulator of homeostatic equilibrium *in vivo*, has been described in antimicrobial host defense mechanisms [81], various pathologies, e.g. cancer [82] and as a defense mechanism against noxious xenobiotics, e.g. venoms [28].

### **Effect of *BG* venom on gene expression**

**Figure 4.**
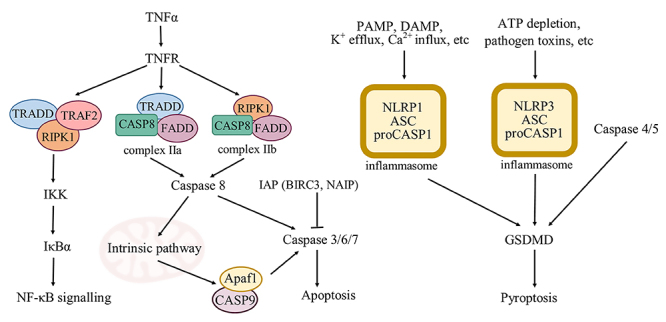
Representative apoptosis-related genes and pathways activated by *BG* venom in 1889c cells. TNF: tumor necrosis factor; TNFR: tumor necrosis factor receptor; TRADD: TNFR associated death domain; TRAF: TNFR associated factor; RIPK: receptor-interacting protein kinase; IKK: inhibitor of nuclear factor kappa-B kinase; NF-κB: nuclear factor kappa-light-chain-enhancer of activated B cells; FADD: Fas-associated death domain; APAF: apoptotic protease activating factor; IAP: inhibitor of apoptosis protein; NLRP: NLR family pyrin domain containing protein 1; GSDMD: gasdermin D; BIRC: baculoviral IAP repeat containing protein; NAIP: NLR family apoptosis inhibitory protein; ASC: caspase recruitment domain; PAMP: pathogen-associated molecular pattern; DAMP: damage-associated molecular pattern.

Members of the NLR family, e.g. NLRP1, NLRP3, are implicated in the recruitment of inflammatory caspases to the inflammasome [83]. Recent evidence published by Youm et al. [84] provided new insights of NLRP induced inflammasome formation and its role in the thymus. Immune-senescence and thymic cell death may be delayed by blocking the pathway resulting in inflammasome production, as observed in NLRP3^-/-^ mice. Our findings suggest that pyroptosis driven inflammasome production, and not classical apoptosis, might be the main cause of cell demise. Significant up-regulation of *NLRP1* gene (15.52-fold) along with genes encoding inflammatory caspases *CASP4* (2.47-fold) and *CASP5* (2,450,677-fold increase) in thymic carcinoma cells corroborate with the findings of Youm et al. [84] and suggest an alternative PCD pathway triggered by *BG* venom in thymic cells (Figure 4).

Caspase-mediated apoptosis induced by different snake venom fractions, for example LAAOs, has been observed by Alves et al. [85]. The apoptotic potential of *Bothrops atrox* LAAOs has been correlated with increased levels of caspase-3 and caspase-9 in HL60, Jurkat, PC12 and B16F10 cell lines [85]. In a communication disclosed by Santos et al. [86] caspase-3 mediated apoptosis of rabbit skeletal muscle myocytes has been attributed to envenomation with *Bothrops alternatus*. Tavares et al. [87] have shown that *Calloselasma rhodostoma* SV-LAAOs might contribute to cytotoxic cellular events by the activation of caspase-3 and caspase-8 in hyperproliferative myeloid cells. In another study, published by Costal-Oliveira et al. [88] an LAAO from *Bothrops atrox* has been shown to contribute to autophagy-induced apoptosis by increasing the oxidative stress in the immediate microenvironment or by triggering various intracellular substrates of keratinocytes. Snake venom LAAOs show diversified metabolic activities that elicit various pro-apoptotic pathways, mainly through the elevation of caspase activity and through oxidative stress. 

Increased caspase activity produced by envenomation with the snake venoms from the Viperidae family seems to be characteristic. In a study presented by Shahbazi et al. [4] the cytotoxic effect of *Pseudocerastes persicus* has been observed in normal fibroblast cells (Hu02) and lung cancer cells (A549) through the activation of caspase-3 and caspase-9. HPLC fractioning of crude snake venom revealed that fraction 21 activated the intracellular apoptotic pathways, putatively belonging to the class of SVMPs. In this view, SVMP fractions present in the BG venom might explain the well-marked elevation of caspases in thymic epithelial cells. 

Activation of cell apoptosis through the main effector caspase molecules is characteristic to venoms of animal origin. In a recent article published by Ceremuga et al. [89] the apoptotic activity of melittin, a bee venom fraction, has been correlated with elevated caspase-3 and caspase-7 levels in a dose- and time-dependent manner. Boeno et al. [90] have shown that purified Lys49-PLA2 homologue from *Bothrops jararacussu* might contribute to inflammasome formation through the activation of NLRP3, which in turn interacts with caspase-1, leading to an inflammatory response via IL-1β. PLA2 mediated inflammatory response was observed by Cedro et al. [91]. Using purified Asp49-PLA2 (5, 10 and 20 μg/mL) from *Bothrops jararaca* snake venom, an increased level of IL-6, IL-1β and PGE2 was described through neutrophil stimulation, although its cytotoxic activity is inferior to those observed in case of the Lys49-PLA2 homologue structure. NLRP3 inflammasome formation and subsequent secretion of inflammatory cytokines was also described by Zoccal et al. [92] in mice envenomed with *Tityus serrulatus* scorpion venom.

Representatives of the TNF superfamily are enrolled in the activation of both pro- and anti-apoptotic cell mechanisms. On one hand, TNFs induced extrinsic apoptosis via TRADD/FADD activated caspase-8/caspase-10, through caspase-3,-6,-7. Apoptosis regulator BAX (BAX)/Bcl2-associated agonist of cell death (BAD)/phorbol-12-myristate-13-acetate-induced protein 1 (NOXA) represents an important regulatory pathway in cells, leading to ripoptosome (complex IIa or complex IIb) formation as a result of FADD/caspase-8 interaction. On the other hand, TNFs are prone to activate anti-apoptotic mechanisms, promoting RIPK-1 ubiquitination, thus marking it for proteosomal degradation, and subsequently inhibiting the RIPK-1-mediated recruitment of FADD and caspase-8 [93]. Our measurements showed an indicative trend towards down-regulation of the genes encoding effector molecules participating in the pro-apoptotic events inside thymic epithelial cells, namely *FADD* (0.68-fold decrease) and *TRADD* (0.62-fold decrease), and no significant increase in the level of *CASP8*. This observation is in accordance with current consensus on cell death [94], stating that in “type I cells” caspase-8 mediated apoptosis cannot be inhibited by over-expression of Bcl-2 family members. This indicates that not apoptosis, but rather PCD may lead to the massive death of thymic epithelial cells. Moreover, recent studies confirmed negative regulation of caspase-10 on caspase-8, thereby inhibiting cell death. Furthermore, the study presented by Horn et al. [95] associates a role in cell survival to caspase-10 by favoring NF-κB signaling via RIPK-1 activation and subsequent IκBα phosphorylation. Our results are in harmony with these observations, as an increase in both *CASP10* (2.30-fold increase) and *RIPK1* (1.53-fold increase) genes is observed, which may explain the elevated levels of survival genes activated through NF-κB signaling. 

Mutual interaction between members of the TNF superfamily and Bcl-2 family is also of key importance in the regulation of cell fate. Under apoptotic stimuli, anti-apoptotic events are prone to exacerbate in order to overcome the noxiousness of these extracellular impacts. Protective effects of BCL-2 under TRAIL-induced apoptosis was demonstrated by Sun et al. [96]. Snake venoms have been reported to contribute to the activation of the cytokine network and induce intracellular inflammatory processes [44]. Similarly, in the present study, a tendency towards over-expression of TNFs was observed. In addition, as a response to inflammatory stimuli, members of the Bcl-family (*BCL2A1* and *BCL2L10)* showed a considerable increase in gene expression (2.88- and 5.08-fold, respectively). Furthermore, although TNF-mediated caspase activation is realized via TRADD/FADD, TNF activated and NF-κB-mediated anti-apoptotic gene transcription (Bcl-family members, inhibitors of apoptosis proteins (IAPs)) is alternatively induced via RIPK1 and TNF receptor-associated factor 2 (TRAF2) [97]. In this context, our recent findings indicate that due to a somewhat down-regulated FADD and TRADD, TNF activity shifted towards NF-κB induced anti-apoptotic signaling, putatively as a response mechanism to an existing inflammation due to envenomation. This tendency might also be augmented by the somewhat increased expression of *CHUK* (1.63-fold increase), the gene encoding the inhibitor of nuclear factor kappa-B kinase subunit alpha (IKK-α), thus contributing to the NF-κB mediated signal transduction.

Increased caspase activity is generally related to apoptotic processes [98]. IAPs are molecular structures, which inhibit caspase activity and caspase-mediated apoptosis. Katagiri et al. [99] disclosed a dual regulatory effect of NAIP in macrophages as a response to *Legionella* infection by inhibiting apoptotic events through APAF-1 down-regulation and simultaneous triggering of inflammasome activation. The observation made by Katagiri et al. [99] and the over-expression of *NAIP* gene (2.49-fold increase) under the effect of *BG* venom suggests that NAIP is potentially contributing to the anti-apoptotic regulatory mechanisms inside cells, representing a viable explanation for the metabolic shift favoring pyroptosis under the tested conditions. Moreover, *BIRC3* gene, encoding a key IAP in the regulation of cell death showed a 3.40-fold increase. This observation further emphasizes cellular communication towards NF-κB, as BIRC3 activation is NF-κB dependent [100].

Caspase-14 activity was identified in epithelial differentiation, being activated independent of apoptotic or inflammatory stimuli [101]. Its main biological function lies in the differentiation of keratinocytes of the epidermis, but caspase-14 activity was also described in the Hassall’s bodies of the thymus [102]. Novel findings describe caspase-14 as a phylogenetically closely related protein to pyroptosis initiator caspases [103]. Recent advances in the elucidation of the anatomic characterization and metabolic role of Hassall’s bodies reveal a heterocellular anatomic structure, consisting of thymic epithelial cells, macrophages, dendritic-, myoid-, mast cells and lymphocytes suggesting a role in lymphopoiesis [104]. In this view, increased gene expression of *CASP14* (9.40-fold) might be the result of a characteristic metabolism of thymic epithelial cells, similar to that previously reported in other epithelial cells.

### **Effect of *DA* venom on gene expression**

**Figure 5.**
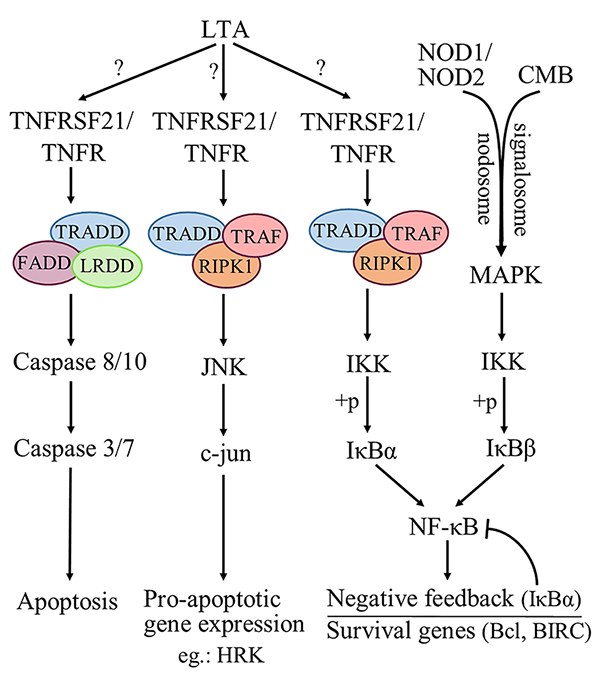
Representative apoptosis-related genes and pathways activated by *DA* venom in human thymic epithelial cells. LTA: lymphotoxin α; TNFR: tumor necrosis factor receptor; TRADD: TNFR associated death domain; FADD: Fas-associated death domain; RIPK: receptor-interacting protein kinase; LRDD: leucine-rich repeats and death domain; MAPK: mitogen activated protein kinase; TRAF: TNFR associated factor; HRK: harakiri; IKK: inhibitor of nuclear factor kappa-B kinase; NF-κB: nuclear factor kappa-light-chain-enhancer of activated B cells; JNK: c-Jun N-terminal kinase; NOD: nucleotide-binding oligomerization domain; CMB: CARD-BCL-MALT complex; Bcl: B-cell lymphoma; BIRC: baculoviral IAP repeat containing protein.

In contrast to the observations made regarding the effect of *BG* venom, in the case of *DA* venom the increase of apoptosis related genes is more profound, while other inflammatory, or pyroptosis initiator members are generally silenced (Figure 5). In contrast to *BG* venom, under the noxious effect of *DA* venom there is a notable down-regulation of *CASP1* (RQ = 0.30). The lack of inflammatory response, leading to inflammasome formation, is also sustained by the down-regulated *TNF* (RQ = 0.59). The correlation between TNF-α and caspase-1 is of key importance during necroptosis, as several studies described a direct activation of caspase-1 by TNF-α [105-107].

From another perspective, the 1.5-fold increase of *TNFRSF21* (encoding DR6), represents a tendency towards TNF-induced apoptosis in thymic epithelial cells. DR6 was identified as a receptor regulating various cellular events through c-Jun N-terminal kinase (JNK) and NF-κB signaling, being expressed in most cell types, including the thymus [108,109]. Recently, Etemadi et al. [110] reported that lymphotoxin α (LT-α) can induce apoptosis and necroptosis in a similar manner as TNF-α by different intracellular cell-fate mechanisms. The marginal elevation of both *TNFRSF21* and *LTA* (1.69-fold) genes may shed novel insight on TNF receptor-induced cell survival. To date, no representative activators of DR6 have been identified. Based on our results, we propose that LT-α, through DR6, contributes to two distinct cell-fate mechanisms. 

On one hand, this ligand-receptor complex can induce apoptosis by activating FADD and TRADD. This is in part confirmed by our study, as *FADD* and the gene encoding its adaptor protein *LRDD* are elevated to some extent (2.39-fold and 1.55-fold, respectively), but *TRADD* presented negligible change in gene expression (1.16-fold). Also, despite the fact that *FADD* is significantly over-expressed, no other important changes were observed in the levels of initiator and effector caspases downstream in this cascade. On the other hand, TNF-receptors contribute to apoptosis via JNK signaling, as downstream of JNK activation pro-apoptotic genes are primarily expressed, e.g. HRK, which presents a significant, 1.86-fold increase in our case. Finally, TNF-receptors are able to activate IKKs, resulting in NF-κB activation and gene transcription of various survival genes. Under the effect of *DA* venom, anti-apoptotic survival genes *BCL2L10* and *BIRC7* showed a noticeable up-regulation of 2.30-fold and 6.47-fold, respectively. Other studies described the activation of both canonical and non-canonical NF-κB pathways by LTs via classical lymphotoxin beta receptor activation [111,112], which might support this dual effect of LT-driven cell fate mechanisms. FADD-independent, MAPK-activated and ERK inactivated apoptotic stimuli have been described by Liu et al. [113] on human leukemia U937 cells following treatment with protease inhibitor-like protein-1 (PILP-1) obtained from *Bungarus multicinctus* snake venom.

As NF-κB is a self-regulatory communication system, under powerful activator stimuli, it also induces the inhibitors of NF-κB, including IκBα (*NFKBIA)* [114]. This would explain the 1.98-fold increase in the *NFKBIA* gene expression in our study.

TRAIL-linked apoptosis, through an over-expression of DR receptors, namely DR4 and DR5 via JNK signaling was described by Park et al. [115] in the presence of *Vipera lebetina turanica* snake venom. Moreover, the tested venom proved to elevate major apoptotic caspases, while the anti-apoptotic proteins, contrary to our results, were depleted. Such differences may be explained by the difference in snake venom composition, as elapid venoms are primarily neurotoxic, while viper venoms are more likely to trigger programmed cell death. Viper snake venoms are generally comprised of SVMPs, SVSPs and PLA_2_, whilst elapid venoms are dominated by PLA_2_ and 3FTxs [116]. Conlon et al. [47] have described the cytotoxic activity of two peptides belonging to the 3FTx superfamily isolated from *D. angusticeps* venom, C13S1C1 which proved to be cytotoxic against breast and colorectal adenocarcinoma cells, and toxin F-VIII which proved to be less potent than the C13S1C1 fraction. Furthermore, several studies elucidate the anticancer activity of snake venoms in various carcinoma cell lines. *Elapidae* venoms are prone to activate effector caspases, such as CASP3 in carcinoma cell lines, and are also prone to induce oxidative stress, or to disrupt the oxidative phosphorylation, and thus cut off the energy household [15,117-119]. 

CARDs are known as protein interaction modules, implicated in protein interactions required for apoptosis and immunity [120]. The CARD9 signalosome is known to mediate NF-κB and mitogen-activated protein kinase (MAPK) signaling, although the exact mechanisms underlying this activation process and the outcome of these molecular interactions are yet to be elucidated [121]. Similarly, recent studies describe the majority of CARDs as NF-κB activators, via the formation of several signaling complexes, like nodosomes (NOD1 and NOD2) and CMB (CARD-BCL-MALT complex), having a regulatory effect over the aforementioned pathways [122]. In our study expression of NF-κB-activator CARDs was inconclusive, as *CARD9* showed a 4.13-fold increase, while *CARD6* and *CARD15* were notably suppressed showing 0.49- and 0.40-fold expression, respectively.

## Conclusion

Our work draws attention to the possible effect of snake envenomation at the level of sub-cellular pathways within the thymic epithelium. Biological toxins (e.g. snake venoms tested here from *B. gabonica* and *D. angusticeps*) can potentially trigger immune senescence of the central immune system (thymus) through apoptotic pathways (e.g. pyroptosis). The subsequent sudden loss in fresh naive T-cell production may lead to elevated chances of secondary immune pathologies such as infection, cancer and auto-immunity, later in life. This not only hampers quality of life, but also poses significant burden on the healthcare and social system, supporting our research.

## Supplementary material

The following online material is available for this article:

Additional file 1. Cell confluence assessment by microscope image analysis (ImageJ software 1.51j8) for representative images presented in Additional file 2.

Additional file 2. Representative microscope images of 1889c cell cultures following **(A-C)** 2-hour or **(D-F)** 24-hour incubation with snake venoms. **(A, D)** Untreated cells (negative control); **(B, E)** 10 μg/mL *Bitis gabonica* venom; **(C, F)** 10 μg/mL *Dendroaspis angusticeps* venom.

Additional file 3. Relative quantification (RQ) values measured following the 24-hour treatment of 1889c with either 10 μg/mL *Bitis gabonica* venom or 10 μg/mL *Dendroaspis angusticeps* venom. Untreated cells served as reference (negative control).
