# Supplementary material for: Effect of Bitis gabonica and Dendroaspis angusticeps snake venoms on apoptosis-related genes in human thymic epithelial cells
Source: J Venom Anim Toxins Incl Trop Dis. 2020 Dec 14;26:e20200057. doi: 10.1590/1678-9199-JVATITD-2020-0057 (PMC7745260; doi:10.1590/1678-9199-JVATITD-2020-0057)
Supplement: Additional file 1. [file 1678-9199-jvatitd-26-e20200057-s1.pdf]

J Venom Anim Toxins incl Trop Dis
https://doi.org/10.1590/1678-9199-JVATITD-2020-0057

Supplementary Material to "Effect of *Bitis gabonica* and *Dendroaspis angusticeps* snake venoms on apoptosis related genes in human thymic epithelial cells"

Additional file 1. Cell confluence assessment by microscope image analysis (ImageJ software 1.51j8) for representative images presented in Additional file 2.

Label	Area (px)	Mean	%Area	MinThr	MaxThr
Additional file 2 – Panel A	2145206	126.740	68.194	27	255
Additional file 2 – Panel B	1482002	136.014	47.112	56	255
Additional file 2 – Panel C	1147253	138.211	36.470	46	255
Additional file 2 – Panel D	616072	153.760	19.584	71	255
Additional file 2 – Panel E	986667	140.757	31.365	24	255
Additional file 2 – Panel F	470523	142.961	14.958	58	255
Total area	3145728	37.455	100.000	0	255

* MinThr: minimum threshold, MaxThr: maximum threshold
