# Supplementary material for: Effect of Bitis gabonica and Dendroaspis angusticeps snake venoms on apoptosis-related genes in human thymic epithelial cells
Source: J Venom Anim Toxins Incl Trop Dis. 2020 Dec 14;26:e20200057. doi: 10.1590/1678-9199-JVATITD-2020-0057 (PMC7745260; doi:10.1590/1678-9199-JVATITD-2020-0057)

J Venom Anim Toxins incl Trop Dis
https://doi.org/10.1590/1678-9199-JVATITD-2020-0057

Supplementary Material to "Effect of *Bitis gabonica* and *Dendroaspis angusticeps* snake venoms on apoptosis related genes in human thymic epithelial cells"

Additional file 2. Representative microscope images of 1889c cell cultures following **(A-C)** 2-hour or **(D-F)** 24-hour incubation with snake venoms. **(A, D)** Untreated cells (negative control); **(B, E)** 10 μg/mL *Bitis gabonica* venom; **(C, F)** 10 μg/mL *Dendroaspis angusticeps* venom.

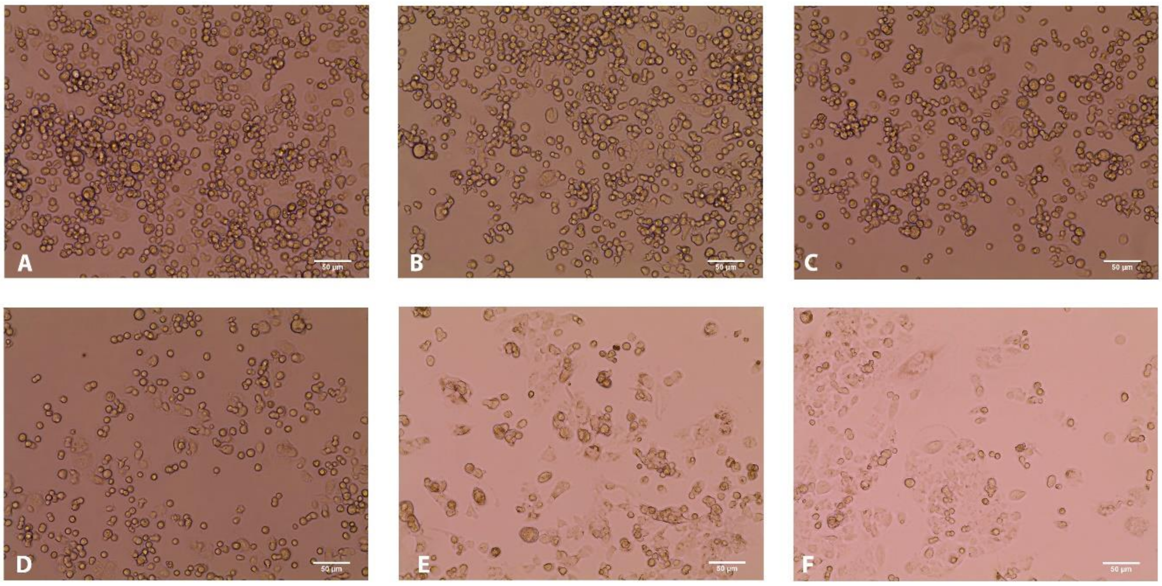

Supplement: Additional file 2. [file 1678-9199-jvatitd-26-e20200057-s2.pdf]
