# Supplementary material for: Effect of Bitis gabonica and Dendroaspis angusticeps snake venoms on apoptosis-related genes in human thymic epithelial cells
Source: J Venom Anim Toxins Incl Trop Dis. 2020 Dec 14;26:e20200057. doi: 10.1590/1678-9199-JVATITD-2020-0057 (PMC7745260; doi:10.1590/1678-9199-JVATITD-2020-0057)
Supplement: Additional file 3. [file 1678-9199-jvatitd-26-e20200057-s3.pdf]

J Venom Anim Toxins incl Trop Dis
https://doi.org/10.1590/1678-9199-JVATITD-2020-0057

Supplementary Material to "Effect of *Bitis gabonica* and *Dendroaspis angusticeps* snake venoms on apoptosis related genes in human thymic epithelial cells"

Additional file 3. Relative quantification (RQ) values measured following the 24-hour treatment of 1889c with either 10 µg/mL *Bitis gabonica* venom or 10 µg/mL *Dendroaspis angusticeps* venom. Untreated cells served as reference (negative control)

Target name	Measured RQ	
	10 µg/mL *BG*	10 µg/mL *DA*
18S [1]	ND	ND
GAPDH [1]	ND	ND
HPRT1 [1]	ND	ND
GUSB [1]	ND	ND
BIRC2	0.958	1.186
APAF1	1.119	0.906
BAD	0.717	1.168
BAK1	0.804	1.468
BAX	0.753	1.261
BBC3	0.875	1.375
BCAP31	0.880	1.180
BCL10	1.515	0.856
BCL2	**2.563**	1.468
BCL2A1	**2.889**	0.861
BCL2L1	1.341	1.135
BCL2L10	**5.080**	**2.303**
BCL2L11	1.051	1.146
BCL2L13	1.282	0.894
BCL2L14	ND	ND
BCL2L2	1.125	1.429
BCL3	0.985	1.153
BID	1.032	1.587
BIK	0.934	0.801
NAIP	**2.495**	1.106
BIRC3	**3.401**	1.325
XIAP	1.323	1.102
BIRC5, EPR1	0.770	1.039
BIRC6	1.256	0.920
BIRC7	ND	**6.469**
BIRC8	ND	ND
BNIP3	1.786	1.251
BNIP3L	**2.079**	1.097
BOK	0.822	1.455
NOD2	1.160	**0.407**
NOD1	1.047	1.053
CARD6	1.375	**0.495**
CARD9	**0.016**	**4.137**
CASP1	0.907	**0.306**
CASP10	**2.299**	1.221
CASP14	**9.405**	ND
CASP2	1.105	1.074
CASP3	1.406	1.037
CASP4	**2.477**	1.137
CASP5	**2,450,677**	ND
CASP6	0.930	0.785
CASP7	1.332	1.126
CASP8	1.442	1.112

https://doi.org/10.1590/1678-9199-JVATITD-2020-0057

**Target name**	**Measured RQ**	
	**10 µg/mL *BG***	**10 µg/mL *DA***
CASP8AP2	1.104	0.966
CASP9	**0.157**	1.298
CFLAR	1.050	1.051
CHUK	1.632	1.085
CRADD	0.897	1.151
DAPK1	0.778	0.763
DEDD	1.334	1.317
DEDD2	1.039	1.441
DIABLO	1.220	1.004
IFT57	0.999	0.818
FADD	0.688	**2.393**
FAS	1.429	1.130
FASLG	ND	ND
HIP1	1.470	1.416
HRK	**0.404**	**1.862**
HTRA2	1.086	1.191
CARD18	0.783	1.056
IKBKB	1.490	1.227
IKBKE	0.919	0.773
IKBKG	0.996	1.042
LRDD	1.001	1.553
LTA	1.245	1.692
LTB	0.561	**0.442**
MCL1	**2.173**	1.399
NLRP1	**15.526**	1.034
NFKB1	1.350	0.929
NFKB2	0.968	0.910
NFKBIA	1.438	**1.980**
NFKBIB	1.238	1.356
NFKBIE	1.255	1.420
NFKBIZ	1.251	0.924
PEA15	1.536	0.770
PMAIP1	1.607	1.192
PYCARD	0.685	1.124
REL	1.357	1.069
RELA	1.637	0.971
RELB	0.939	1.547
RIPK1	1.538	1.211
RIPK2	1.093	0.839
TBK1	1.375	0.902
TNF	1.453	0.598
TNFRSF10A	1.703	1.223
TNFRSF10B	1.404	1.426
TNFRSF1A	1.015	1.102
TNFRSF1B	0.669	0.950
TNFRSF21	1.748	1.595
TNFRSF25	1.339	1.428
TNFSF10	**1.907**	1.489
TRADD	0.619	1.160

[1]Endogenous control genes. ND: not detectable. Significant fold change values are highlighted in bold.
